# Inertial Measurement Unit-Based Estimation of Foot Trajectory for Clinical Gait Analysis

**DOI:** 10.3389/fphys.2019.01530

**Published:** 2020-01-10

**Authors:** Koyu Hori, Yufeng Mao, Yumi Ono, Hiroki Ora, Yuki Hirobe, Hiroyuki Sawada, Akira Inaba, Satoshi Orimo, Yoshihiro Miyake

**Affiliations:** ^1^School of Computing, Tokyo Institute of Technology, Yokohama, Japan; ^2^Department of Neurology, Kanto Central Hospital, Tokyo, Japan

**Keywords:** gait analysis, IMU, abnormal gait, inertial-measurement unit, wearable sensors

## Abstract

Gait analysis is used widely in clinical practice to evaluate abnormal gait caused by disease. Conventionally, medical professionals use motion capture systems or make visual observations to evaluate a patient's gait. Recent biomedical engineering studies have proposed easy-to-use gait analysis methods employing wearable sensors with inertial measurement units (IMUs). IMUs placed on the shanks just above the ankles allow for long-term gait monitoring because the participant can walk with or without shoes during the analysis. To the knowledge of the authors, no IMU-based gait analysis method has been reported that estimates stride length, gait speed, stride duration, stance duration, and swing duration simultaneously. In the present study, we tested a proposed gait analysis method that uses IMUs attached on the shanks to estimate foot trajectory and temporal gait parameters. Our proposed method comprises two steps: stepwise dissociation of continuous gait data into multiple steps and three-dimensional trajectory estimation from data obtained from accelerometers and gyroscopes. We evaluated this proposed method by analyzing the gait of 19 able-bodied participants (mean age 23.9 years, 9 men and 10 women). Wearable sensors were attached on the participants' shanks, and we measured three-axis acceleration and three-axis angular velocity with the sensors to estimate foot trajectory during walking. We compared gait parameters estimated from the foot trajectory obtained with the proposed method and those measured with a motion capture system. Mean accuracy (± standard deviation) was 0.054 ± 0.031 m for stride length, 0.034 ± 0.039 m/s for gait speed, 0.002 ± 0.020 s for stride duration, 0.000 ± 0.017 s for stance duration, and 0.002 ± 0.024 s for swing duration. These results suggest that the proposed method is suitable for gait analysis, whereas there is a room for improvement of its accuracy and further development of this IMU-based gait analysis method will enable us to use such systems for clinical gait analysis.

## Introduction

Analysis of abnormal gait can provide important information about diseases and injuries. For example, patients with Parkinson's disease (PD) often exhibit shuffling, festinating, and freezing of gait. The most widely used clinical rating scale for PD, the Unified Parkinson's Disease Rating Scale, includes observation of gait (Goetz et al., 2008). Patients with cerebellar disorders sometimes have a wide-based (atactic) gait, and those with cerebral vascular disease sometimes exhibit a hemiplegic gait. Recent articles have reported changes in gait, such as reduced gait velocity and stride length, in diseases with gait disorders and in other conditions, such as Alzheimer's disease (Mielke et al., 2013) and depression (Lemke et al., 2000).

Clinical gait analysis is performed mostly by health-care providers using visual observation (Krebs et al., 1985). Although this method is the most readily accessible means of gait analysis available to health-care providers (Barker et al., 2006), it is a subjective and qualitative method that is inadequate for assessing changes in gait features during ongoing treatment interventions. It is also difficult for clinicians to share this information with health-care providers and patients. Motion capture systems are used in clinical research for gait analysis (Mcginley et al., 2009) and scientific research (Lieberman et al., 2010). Because they provide well-quantified and accurate results, these systems are currently considered to be the criterion standard for clinical gait analysis (Cameron and Wagner, 2011). However, because the special equipment needed for motion capture is expensive and requires a large space, few medical institutions can use these systems for clinical gait analysis (Barker et al., 2006).

Several studies have proposed gait analysis methods using inertial measurement units (IMUs) to solve the problems described above (Sabatini et al., 2005; Moore et al., 2007; Mariani et al., 2010; Rebula et al., 2013; Kitagawa and Ogihara, 2016). IMUs used in these methods are inexpensive and wearable (Fujiki et al., 2009). In particular, we focused on methods that estimate trajectories of a foot because such methods can be used to obtain several spatial gait parameters. Sabatini et al. (2005) proposed an IMU-based gait analysis method that estimates a two-dimensional trajectory in the sagittal plane of a foot during walking. Other studies have proposed gait analysis methods that estimate the three-dimensional foot trajectory during walking in a stepwise manner to obtain values of foot clearance (Mariani et al., 2010, 2012; Kitagawa and Ogihara, 2016). The trajectory estimation methods reported in several studies (Sabatini et al., 2005; Mariani et al., 2012; Kitagawa and Ogihara, 2016) use an IMU attached on the dorsum of the foot and are better for obtaining this gait feature. As described above, and to the knowledge of the authors, there is no report of a method that estimates three-dimensional foot trajectory from an IMU attached on the shank during waking in a stepwise manner to calculate simultaneously spatial and temporal clinical gait parameters, including stride length, gait speed, stride duration, stance duration, and swing duration.

In this study, we propose a novel gait analysis method for clinical purposes that uses IMUs attached on the shanks to estimate foot trajectory and to obtain estimated clinical gait parameters. We conducted an experimental evaluation of the proposed method. Here, we report an example of the application of the proposed method to PD patients.

## Proposed Method

### Sensors Used and Wearing Method

**Figure 1:**
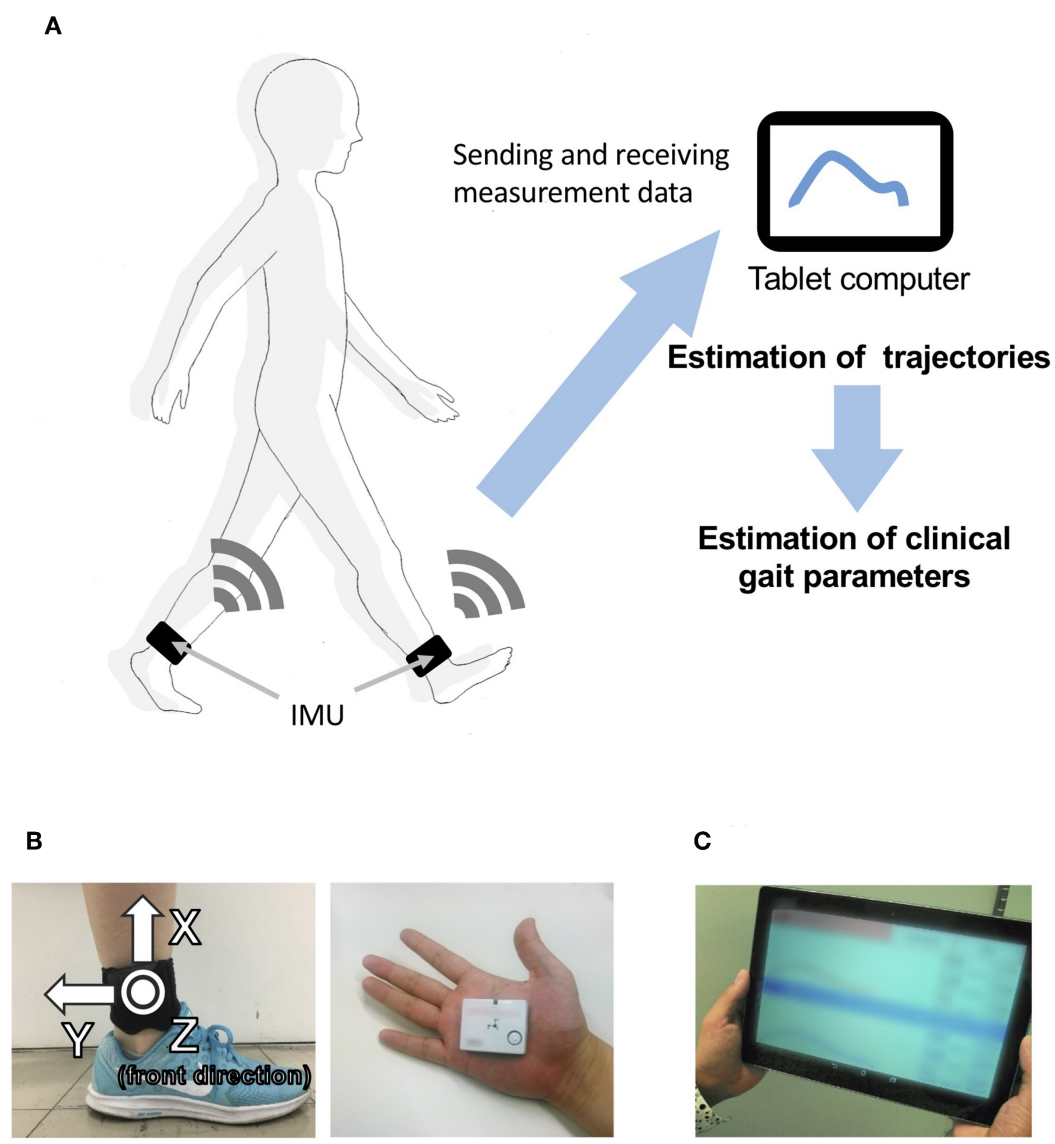
Overview of the system. **(A)** System configuration in the proposed method, including **(B)** a wearable sensor and its attachment on the shanks (just above the ankles), and **(C)** a tablet computer.

Our proposed gait analysis system is illustrated in Figure 1A. For gait analysis, we used two IMUs (TSND121, ATR-Promotions, Kyoto, Japan; Figure 1B) with a triaxial accelerometer (±8 G range), triaxial gyroscope (±1,000° per range), and Android OS tablet (ZenPad10, ASUSTeK Computer Inc., Taipei, Taiwan; Figure 1C). Raw accelerometer and gyroscope signals were sampled at 100 Hz (16 bits per sample). The size of the IMU is 37 mm × 46 mm × 12 mm and its weight is about 22 g. IMUs are attached on the shanks (just above the ankles) with bands (Figure 1B). The inertial coordinate system used to represent foot orientation and position is shown in Figure 1B. Acceleration and angular velocity data of both the shanks measured during walking are transmitted to the tablet through Bluetooth.

### Algorithm for Trajectory Estimation

Our proposed method comprises two steps: dissociation of continuous gait data into multiple steps and three-dimensional trajectory estimation in a stepwise manner. Each process is described as follows.

### Stepwise Dissociation From Angular Velocity Signals

We dissociated the signals into four steps: (1) smoothing and finding the local maximums; (2) finding the heel-strike (HS) and toe-off (TO) points; (3) quadratic regression; and (4) calculating the split point. One walking cycle is defined as a single step, and the starting point of each cycle is defined as the split point between HS and immediate TO points. We identify the split point in each cycle based on raw angular velocity data in the *z*-axis ω_*z*_ of the gyroscope (right-handed; Figure 1B, left).

#### Smoothing and Finding the Local Maximums

**Figure 2:**
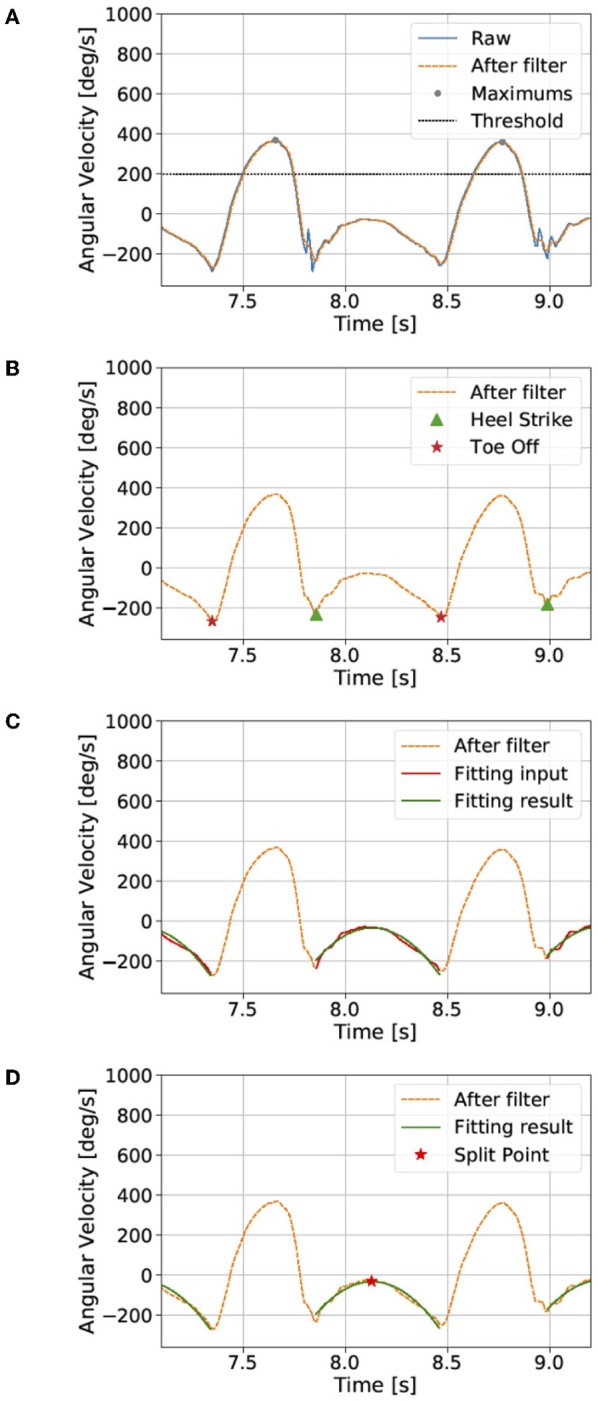
Dissociation of the continuous gait signal (see the section Proposed method). The raw data contains much noise, and a median filter was first used to smooth the data. **(A)** We found local maxima that are larger than a threshold and **(B)** then the heel-strike and toe-off points. **(C)** We assume a quadratic curve between the heel-strike and toe-off points. **(D)** Finally, the split point was defined as the maximum point of the quadratic fitting result. The gait cycle was defined by the data from the split point to the next split point.

The raw data contained much noise, and a median filter (window length: 5) was first used to smooth the data. We found the local maximum *l*_*k*_ that is larger than the threshold (200°/s; Figure 2A).

#### Finding the Local Minimums Near the HS and TO Points

We then found the *k*-th local minimums near HS *mhs*_*k*_ and TO *mto*_*k*_ points between the *l*_*k*_ and *l*_*k*+1_. *mhs*_*k*_ is the local minimum closest to the right of *l*_*k*_, and *mto*_*k*_ is the local minimum closest to the left of *l*_*k*+1_ (Figure 2B).

#### Quadratic Regression

We assume ω_*z*_ between *mhs*_*k*_ and *mto*_*k*_ points to represent a quadratic curve (Figure 2C):

(1) $$\begin{array}{l} \begin{array}{c} {\hat{\omega }}_{z,t}=a_{k}t^{2}+b_{k}t+c_{k},\;t\in \left[mhs_{k},\;mto_{k}\right], \end{array} \end{array}$$

where ${\hat{\omega }}_{z,t}$ is the best-fitting result for angular velocity in the *z*-axis.

A quadratic regression is then used to fit the raw data and to calculate the parameters *a*_*k*_, *b*_*k*_, *c*_*k*_:

(2) $$\left[\begin{array}{lll} \sum t^{4} & \sum t^{3} & \sum t^{2}\\ \sum t^{3} & \sum t^{2} & \sum t\\ \sum t^{2} & \sum t & M_{k} \end{array}\right]\left[\begin{array}{l} a_{k}\\ b_{k}\\ c_{k} \end{array}\right]=\left[\begin{array}{c} \sum t^{2}\omega_{z,t}\\ \sum t\omega_{z,t}\\ \sum \omega_{z,t} \end{array}\right],$$

where *M*_*k*_ is the number of points between *mhs*_*k*_ and *mto*_*k*_.

#### Calculating the Split Point

Finally, the segmentation point *sp*_*k*_ was defined as the maximum point of the quadratic fitting result (Figure 2D):

$$\begin{array}{l} sp_{k}=\underset{t}{\text{argmax}}\left({\hat{\omega }}_{z,t}\right),\;t\in \left[mhs_{k},\;mto_{k}\right]. \end{array}$$

The *k*-th cycle was defined by the data between *sp*_*k*_ and *sp*_*k*+1_. In each cycle, we estimate the trajectory.

### Estimating the Trajectory of a Step

The foot trajectory in each cycle can be calculated by integrating the acceleration between each segmentation point. Two coordinate systems are applied: a laboratory coordinate system (*e*) and a sensor coordinate system (*s*). Because the raw data from the sensor are represented by the time-variant sensor coordinates, we need to transpose them into time-invariant laboratory coordinates. Acceleration in the laboratory coordinates can be converted from measured sensor acceleration using a rotational matrix:

(3) $$=\begin{array}{c} R_{s\to e}=R_{x}\left(\theta \right)R_{y}\left(\phi \right)R_{z}\left(\psi \right)\\ \left[\begin{array}{ccc} \cos\phi \cos\psi \; & -\cos\phi \sin\psi \; & \sin\phi \\ \sin\theta \sin\phi \cos\psi & -\sin\theta \sin\phi \sin\psi \; & -\sin\theta \cos\phi \\ +\cos\theta \sin\psi & +\cos\theta \cos\psi & \;\\ -\cos\theta \sin\phi \cos\psi & \cos\theta \sin\phi \sin\psi & \cos\theta \cos\phi \\ \; & +\sin\theta \cos\psi & \; \end{array}\right] \end{array},$$

where θ, ϕ, *and ψ* are the Euler angles around the *x-, y-*, and *z*-axes.

We divide the estimation process of the foot trajectory into five steps: (1) calculate the initial Euler angles; (2) calculate the time derivative of the Euler angles; (3) calculate the Euler angles using the integral; (4) transpose the accelerations using a rotational matrix; and (5) calculate the trajectory using the double integral.

#### Calculating the Initial Euler Angles

At the beginning of each cycle, we can assume that the foot is in full contact with the floor and is momentarily stationary. The accelerometer is assumed to detect only gravitational acceleration **g** at the outset:

(4) $$\begin{array}{c} a_{s,0}=\left[\begin{array}{c} a_{x,0}\\ a_{y,0}\\ a_{z,0} \end{array}\right]=R_{s\to e}^{-1}g=\left[\begin{array}{c} -\cos\phi_{0}\cos\psi_{0}\;\\ \cos\phi_{0}\sin\psi_{0}\;\\ -\sin\phi_{0} \end{array}\right], \end{array}$$

where **a**_*s*,0_ is the initial acceleration vector in the sensor coordinate, and ϕ_0_ and ψ_0_ are the initial Euler angles around the *y*- and *z*-axes.

Therefore, the initial Euler angles vector **θ**_0_ can be calculated as:

(5) $$\begin{array}{l} \theta_{0}=\left[\begin{array}{c} \theta_{0}\\ \phi_{0}\\ \psi_{0} \end{array}\right]=\left[\begin{array}{c} 0\\ \tan^{-1}\left(-a_{z,0}/\sqrt{a_{x,0}^{2}+a_{y,0}^{2}}\right)\\ \tan^{-1}\left(-a_{y,0}/a_{x,0}\right) \end{array}\right]. \end{array}$$

#### Calculating the Time Derivative of the Euler Angles

The relation between the *i*th angular velocity **ω**_*i*_ from the gyroscope and the time derivation of Euler angles ${\dot{\theta }}_{i}$ can be calculated as:

(6) $${\dot{\theta }}_{i}=\left[\begin{array}{c} {\dot{\theta }}_{i}\\ \dot{\phi_{i}}\\ {\dot{\psi }}_{i} \end{array}\right]=\left[\begin{array}{ccc} \cos\psi_{i}/\cos\phi_{i} & -\sin\psi_{i}/\cos\phi_{i} & \;0\;\\ \sin\psi_{i} & \cos\psi_{i} & \;0\;\\ -\tan\phi_{i}\cos\psi_{i}\; & \tan\phi_{i}\sin\psi_{i} & \;1\; \end{array}\right]\omega_{i},$$

where ϕ_*i*_ and ψ_*i*_ are the *i*th Euler angles around the *y*- and *z*-axes.

#### Calculating the Euler Angles by Integral

Euler angles are derived by the integration of Euler angle derivation:

(7) $$\begin{array}{l} \begin{array}{c} \theta_{i}=\theta_{i-1}+\theta_{i}\Delta t, \end{array} \end{array}$$

where Δ*t* is the sampling rate.

#### Transposing Accelerations Using a Rotational Matrix

The rotational matrix calculated by Equation (3) is used to estimate the acceleration in laboratory coordinates **R**_*s*→*e*_**a**_*s*_, which contains the gravitational acceleration. Linear acceleration **a**_*e*_ in laboratory coordinates can then be calculated by simply subtracting gravity:

(8) $$\begin{array}{l} \begin{array}{c} {\text{a}}_{e}={\text{R}}_{s\to e}{\text{a}}_{s}-\text{g}. \end{array} \end{array}$$

#### Calculating Trajectory Using the Double Integral

The *i*-th velocity **v**_*e,i*_ in the laboratory coordinates is estimated by integration of linear acceleration **a**_*e,i*_:

(9) $$\begin{array}{l} \begin{array}{c} {\text{v}}_{e,i}={\text{v}}_{e,i-1}+\frac{\left({\text{a}}_{e,i}+{\text{a}}_{e,i-1}\right)}{2}\Delta t, \end{array} \end{array}$$

and the *i*-th foot trajectory **p**_*e,i*_ is estimated by integration of *v*_*e,i*_:

(10) $$\begin{array}{l} \begin{array}{c} {\text{p}}_{e,i}={\text{p}}_{e,i-1}+\frac{\left({\text{v}}_{e,i}+{\text{v}}_{e,i-1}\right)}{2}\Delta t. \end{array} \end{array}$$

### Reducing of Brownian Noise

Integration may drift because of IMU sensor error, and the calculations of velocity and trajectory are corrected by the constraint condition. In each cycle, both the initial value and the end value of the velocity in three directions and the trajectory in the vertical direction can be assumed as 0. The algorithm below shows how to estimate velocity.

First, the forward integral and back integral are calculated separately from linear acceleration **a**_*e,i*_:

(11) $$\begin{array}{l} \begin{array}{c} {\text{v}}_{e,i}^{f}={\text{v}}_{e,i-1}^{f}+\frac{\left({\text{a}}_{e,i}+{\text{a}}_{e,i-1}\right)}{2}\;\Delta t, \end{array} \end{array}$$

(12) $$\begin{array}{l} \begin{array}{c} {\text{v}}_{e,i}^{b}={\text{v}}_{e,i+1}^{b}+\frac{\left({\text{a}}_{e,i}+{\text{a}}_{e,i+1}\right)}{2}\;\Delta t, \end{array} \end{array}$$

where the superscripts *f* and *b* mean forward and backward, respectively. The correction result can then be derived by the weighted average of the forward and backward integral:

(13) $$\begin{array}{l} \begin{array}{c} {\text{v}}_{e,i}={w_{i}\text{v}}_{e,i}^{f}+\left(1-w_{i}\right){\text{v}}_{e,i}^{b}, \end{array} \end{array}$$

where *w*_*i*_ is the weight and *w*_*i*_ ∈ [0, 1].

Because ${\text{v}}_{e,i}^{f}$ is more accurate near the starting point and ${\text{v}}_{e,i}^{b}$ is more accurate near the end point, the function for calculating *w*_*i*_ should increase monotonically. Here, we choose the sigmoid function to calculate *w*_*i*_:

(14) $$\begin{array}{l} \begin{array}{c} w_{i}=\frac{1}{1+\exp\left(m\left(i-\frac{N}{2}\right)\right)}, \end{array} \end{array}$$

where *N* is the number of points in the current cycle and *m* is a hyperparameter calculated from experiment, which we choose as *m* = 0.1. The trajectory in the vertical direction can also be calculated using the algorithm above.

### Estimating of Spatial and Temporal Parameters for Clinical Gait Analysis

The gait events included the HS and TO points, which were extracted first. The HS and TO events detection algorithm was based on the peak detection of the raw angular velocity in sagittal plane ω_*z*_. At the end of the swing period, several of negative peaks can be observed in ω_*z*_ and the first one is associated with the HS instant (Salarian et al., 2004). Before the swing period, a negative peak is associated with the TO instant (Salarian et al., 2004).

For the definition of each gait event search region, the pattern of shank tilt angle, which was inspired from an instep-based previous study (Tunca et al., 2017) was introduced. At the end of the swing period, the shank will rotate around the knee axis caused by knee extension and reach the maximum forward. Then, the rotation of the shank around the ankle axis will start and the foot contact with the ground will produce the HS instant. In this process, θ_*z*_ will appear to increase first and then decrease. Thus a positive peak will appear in θ_*z*_ before the HS instant where θ_*z*_ was computed via the integration of ω_*z*_with the sampling interval Δ*t*. For the convenience of the description, we refer to this instant where peak occurrence as shank-max-forward (SMF). Similarly, after the TO instant, the ankle will lift slightly and rotate until reaching a certain height. Then it will start to rotate and present a negative peak in θ_*z*_. We refer to this instant as the shank-max-backward (SMB) for convenience. As a result, SMF and SMB of θ_*z*_ can be used to define a proper search interval of the HS and TO. We found the SMF and SMB via a peak search algorithm *signal.find_peaks* in SciPy (version 1.2.0) which can find proper peaks via the prominence (define intrinsic height of a peak) and the distance (define the distance between peaks) properties. Then, SMFs are the positive peaks and SMBs are the negative peaks whose prominence is larger than 0.2 [rad] and the distance is at least 0.4 [s]. Finally, HS is defined as the first peak appears after each SMF instant, and the TO is the minimum of angular velocity in the interval (SMB − 0.3 [s], SMB).

Estimated stride length *SL* is calculated by the trajectory in the *y* and *z* direction:

(15) $$\begin{array}{l} \begin{array}{c} SL=\sqrt{{\left(p_{y,N-1}-p_{y,0}\right)}^{2}+{\left(p_{z,N-1}-p_{z,0}\right)}^{2}}, \end{array} \end{array}$$

where ${\text{p}}_{e,i}={\left(p_{x,i}{,p}_{y,i},p_{z,i}\right)}^{T}$. Estimated stride duration is calculated as the time from one HS to the next ipsilateral HS. Estimated gait speed is calculated as the value obtained by dividing stride length by stride duration. Estimated stance duration is calculated as the time from HS to ipsilateral TO. Estimated swing duration time is calculated as the time from TO to the next ipsilateral HS.

## Evaluation of the Proposed Method

### Overview of the Evaluation

We conducted an experimental evaluation of our proposed method to validate the accuracy of the trajectory estimation of the shanks (just above the ankles) to verify whether it allows the analysis of gait for clinical purposes. Twenty healthy people participated in the experiment, and we used a motion capture system as the criterion standard for the evaluating gait in a clinical setting. We evaluated the accuracy of our proposed method for calculating the estimated trajectory and clinical gait parameters. We used an IMU attached on the shanks for the experimental evaluation.

**Figure 3:**
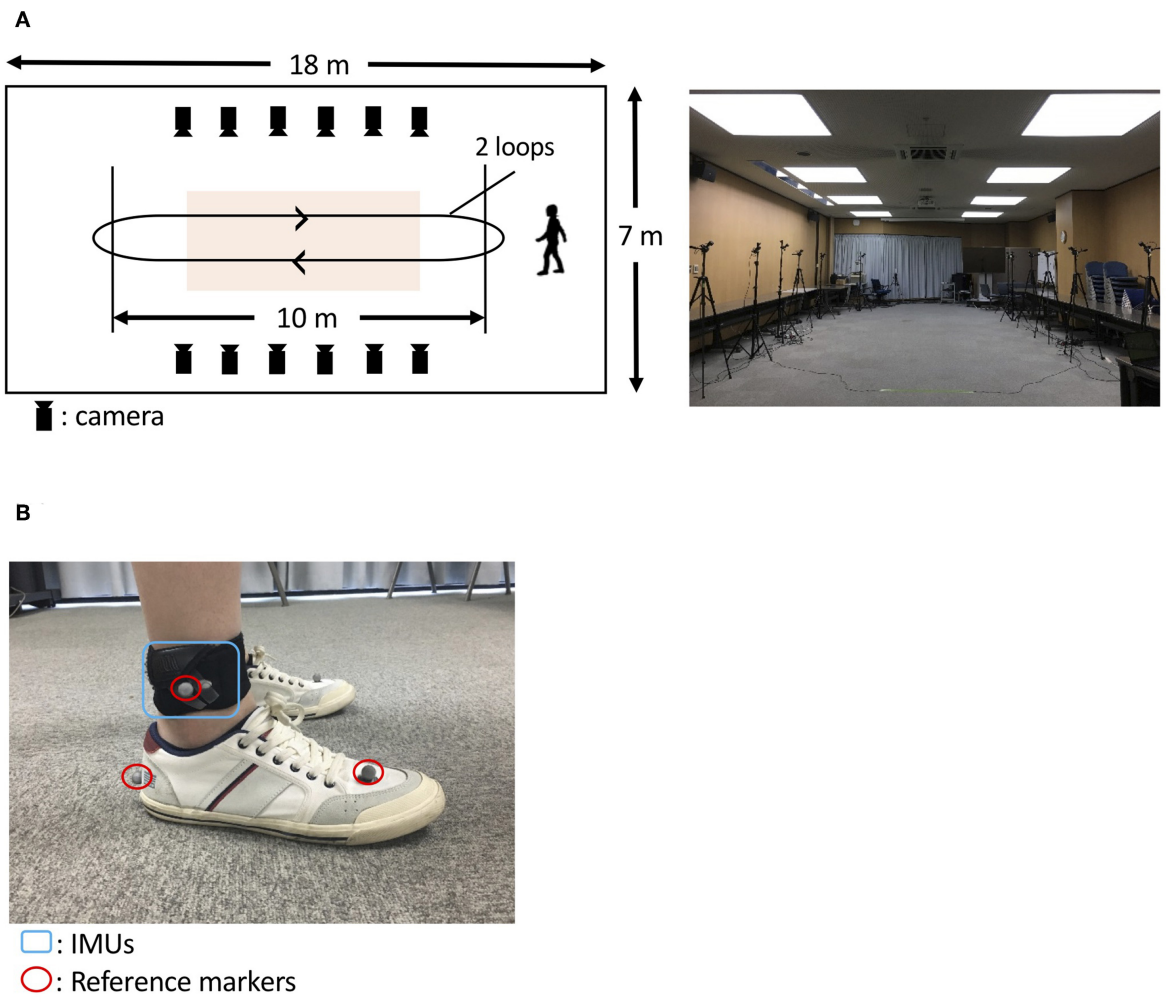
Protocol to evaluate the proposed method. **(A)** A room at the Tokyo Institute of Technology was used (**A**, right). The orange-shaded area shows the measurement area for gait analysis. The participants with IMUs and optical markers **(B)** walked around the room twice, as indicated by the arrows (**A**, left).

An optical motion capture system (Nobby Tech. Ltd., Tokyo, Japan) was used as the reference system. We used 12 cameras, and the motion capture volume was about 2 m × 7 m × 1 m (width, length, and height; Figure 3A). The position error of the markers of the motion capture system during the calibration was <1 mm. The dimensions of the floor of the room were 18 m × 7 m (length and width; Figure 3A). Three optical markers were attached on each foot as shown in Figure 3B. Two of the three markers were attached on the heel and the toe (metatarsal head II) to assess gait parameters. The third marker was attached on the IMU to evaluate the trajectory estimation. To synchronize the IMU and the motion capture system, the participants hit their heel to the floor before the gait measurement. The peaks of the spike waveforms, that were caused by the heel hits of the participants, in both the IMU signals and the motion capture system data were used to define time 0.

### Participants

We recruited 20 able-bodied volunteer participants, with no history of gait abnormalities, from the Tokyo Institute of Technology for the experimental evaluation. The Ethics Committee of Tokyo Institute of Technology approved the protocols for the evaluation, and all participants provided their written informed consent. Because of technical problems with the motion capture system, the data for one of the 20 volunteer participants were excluded from the analyses. Ultimately, we used data from 19 participants (mean age 23.9 years, 9 men and 10 women, mean height 1.66 ± 0.07 m and mean body mass index 20.2 ± 2.7).

As an example of the application of the proposed method to PD patients, we used the method to analyze gait in one healthy elderly participant and four patients with PD. The healthy elderly participant was recruited from a public interest incorporated association in Machida City, Tokyo that provides human resource services for elderly people. Patients with PD were recruited from Kanto Central Hospital, Tokyo. PD had been diagnosed by a physician. The exclusion criteria for this study were past history of other neurological or orthopedic disorders that can affect gait or posture (excluding PD). The healthy elderly participant and the four PD participants provided written informed consent in accordance with requirements of the Ethics Committee of Tokyo Institute of Technology. The Kanto Central Hospital Ethics Committee and the Ethics Committee of Tokyo Institute of Technology approved the protocol for the application of the proposed method.

### Experimental Task

To construct the spike waveforms for synchronization between the IMU and the motion capture system (for the synchronization method, see “Overview of the evaluation”), the participants hit their heel to the floor before the gait measurement. In two trials, the participants walked on a flat floor at their own self-selected natural pace and a slow pace. In each trial, the participants walk straight in the motion capture volume and turned outside of the motion capture volume. Thus, each trial comprised four straight walks (two round trips; Figure 3A, left). We used the gait data obtained as the participants walked in the motion capture volume and removed the gait data as the participants turned outside of the motion capture volume.

### Validation of the Location of IMUs

To consider the validity of the estimation of foot trajectory from IMUs attached on the shanks, we calculated correlations between stride length estimated with the proposed method, measured with a motion capture marker attached on the IMU, and measured with a motion capture marker attached on the heel.

## Results

To evaluate the proposed method, the shank (just above the ankle) trajectory and clinical gait parameters calculated by our proposed method were compared with those collected by the motion capture system. The comparisons of trajectory information were conducted in the sagittal plane. Five clinical gait parameters (stride length, gait speed, stride duration, stance duration, and swing duration) were compared.

### Comparison of the Trajectory Estimated With the Proposed Method and the Motion Capture System

**Figure 4:**
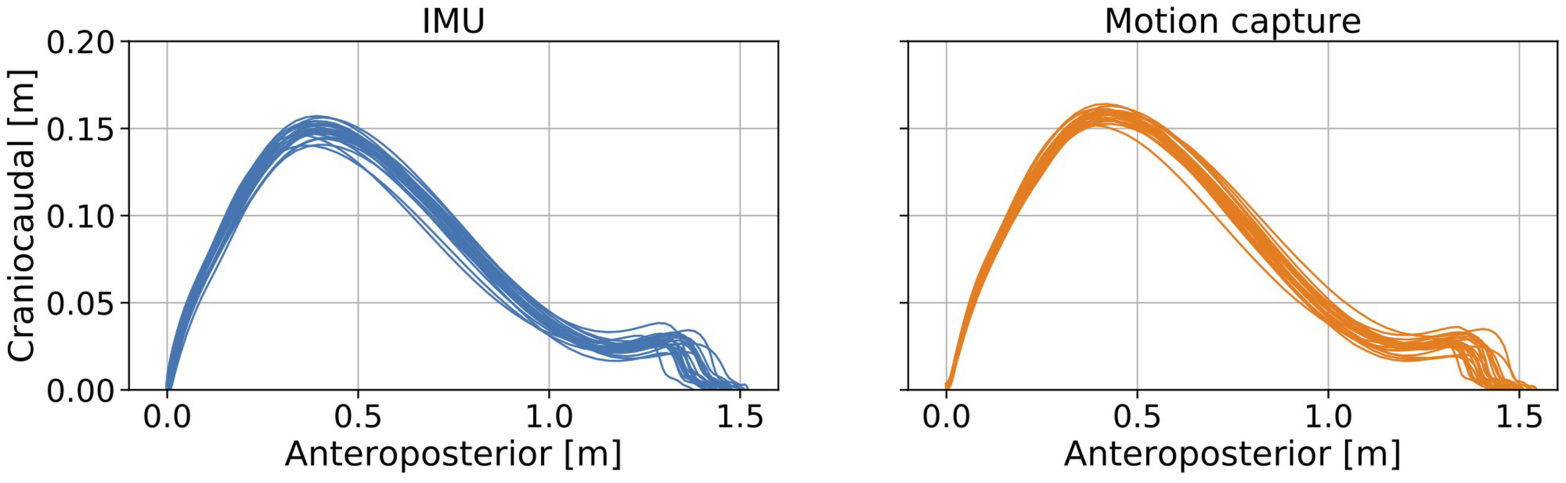
Errors between the estimated foot trajectory identified with our proposed method and reference data from the motion capture system projected in the sagittal plane.

**Figure 5:**
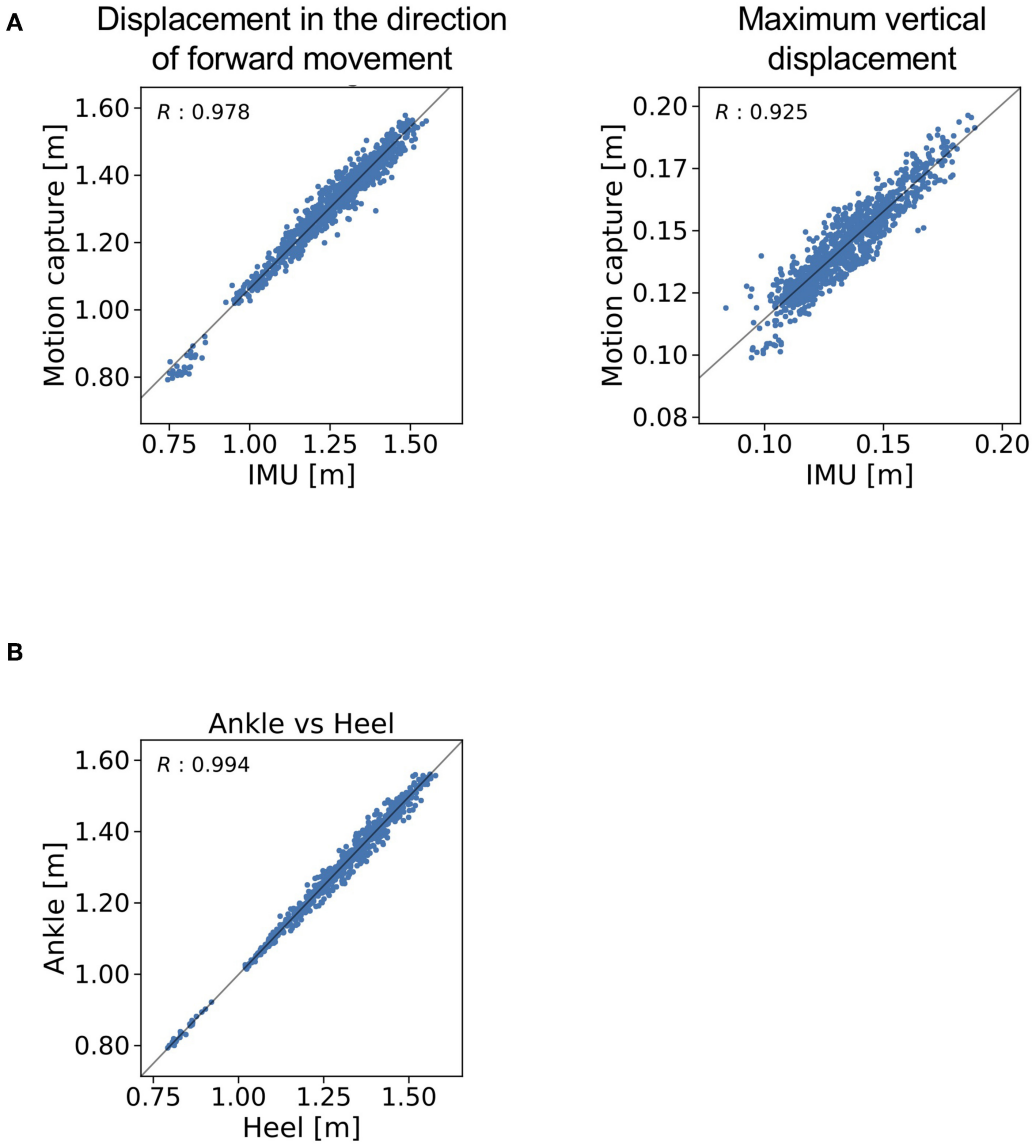
R values for **(A)** the displacement in the direction of forward movement and maximum vertical displacement estimated with the proposed method and **(B)** measured with a marker attached on the IMU and measured with a marker attached on the heel.

The trajectories of our proposed method and the reference data are shown in Figure 4. The *R* value between displacement in the direction of forward movement calculated with the proposed method and measured with a marker attached on the IMU was 0.978 (Figure 5A, left). The *R* value between the maximum vertical displacement calculated with the proposed method and measured with a marker attached on the IMU was 0.925 (Figure 5A, right).

### Validation of the Location of IMUs

The R value between displacement in the direction of forward movement calculated with the marker attached on the IMU and that measured with the marker attached on the heel was 0.994 (Figure 5B).

### Estimation of Clinical Gait Parameters

**Figure 6:**
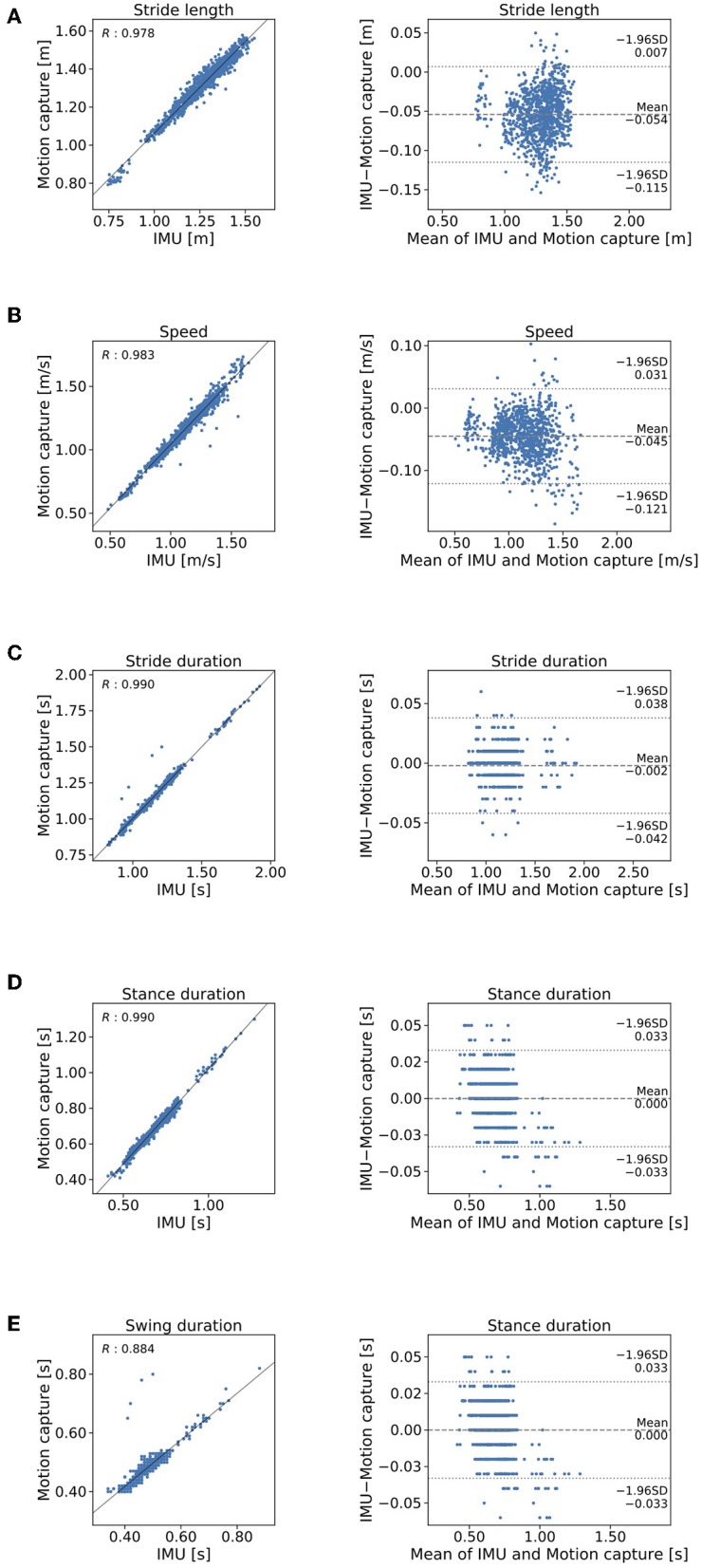
Comparison of the proposed method and the criterion standard in stepwise manner. Mocap, motion capture. Scatterplos and Bland-Altman plots of **(A)** stride length, **(B)** gait speed, **(C)** stride duration, **(D)** stance duration, and **(E)** swing duration.

The means and standard deviations (SD) of the gait parameters compared between the proposed method and the motion capture system are summarized in Table 1. Figure 6 shows the agreement between the proposed method and the motion capture system in Bland-Altman plots. The mean ±1 SD accuracy of stride length was 0.054 ± 0.031 m (Figure 6A). The *R* value between displacement in the direction of forward movement calculated with the proposed method and measured with a marker attached on the heel was 0.978 (Figure 6A). The mean ±1 SD accuracy were as follows: 0.034 ± 0.039 m/s for gait speed (Figure 6B); 0.002 ± 0.020 s for stride duration (Figure 6C); 0.000 ± 0.017 s for stance duration (Figure 6D); and 0.002 ± 0.024 s for swing duration (Figure 6E).

**Table 1:** Comparison between the results of the proposed method and a motion capture system.

	**IMU—Motion capture**	
**Parameter**	**Mean**	**SD**
Stride length [m]	−0.054	0.031
Gait speed [m/s]	−0.034	0.039
Stride duration [s]	−0.002	0.020
Stance duration [s]	0.000	0.017
Swing duration [s]	−0.002	0.024

### Application of the Proposed Method to Patients With a Gait Disorder

**Figure 7:**
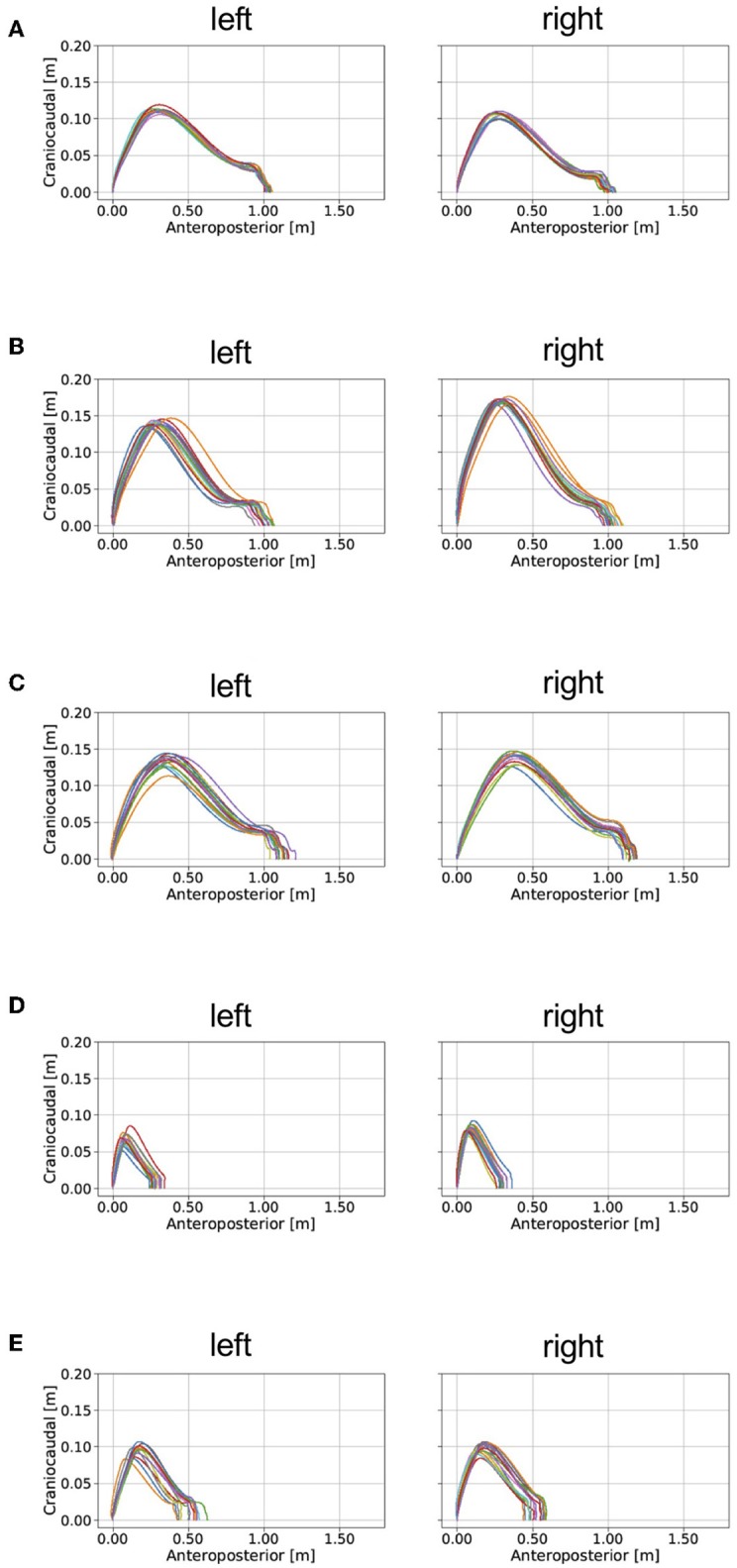
Examples of the application of our proposed method for analyzing gait in patients with PD and a healthy elderly subject. mH&Y, modified Hoehn and Yahr scale. The estimated gait trajectories of **(A)** a healthy elderly subject, **(B)** a patient with PD (mH&Y 2), **(C)** a patient with PD (mH&Y 2), **(D)** a patient with PD (mH&Y 4), and **(E)** a patient with PD (mH&Y 4) are plotted. The left panels shows the trajectory of the left foot, and the right panels shows the trajectory of the right foot.

The shank trajectory over 15 steps for each participant is shown in Figure 7. The mean clinical gait parameters of the PD patients are summarized in Table 2.

**Table 2:** Mean clinical gait parameters of patients with PD.

**Patient ID (mH&Y)**		**Pt1 (2)**	**Pt2 (2)**	**Pt3 (4)**	**Pt4 (4)**
Parameter	Stride length [m]	1.03 (0.04)	1.14 (0.04)	0.29 (0.03)	0.52 (0.05)
	Gait speed [m/s]	0.84 (0.04)	1.05 (0.04)	0.30 (0.02)	0.51 (0.06)
	Stride duration [s]	1.22 (0.04)	1.09 (0.02)	0.97 (0.06)	1.02 (0.05)
	Stance duration [s]	0.71 (0.04)	0.57 (0.03)	0.62 (0.05)	0.62 (0.06)
	Swing duration [s]	0.52 (0.04)	0.52 (0.02)	0.35 (0.04)	0.40 (0.04)

*Values are expressed as mean (standard deviation). mH&Y, modified Hoehn and Yahr scale; Pt, patient*.

## Discussion

We have proposed a new method for gait analysis that uses IMUs attached on the shanks to estimate foot trajectory and then to obtain estimated clinical gait parameters. The gait parameters obtained with the proposed method consists of stride length, gait speed, stride duration, stance duration, and swing duration. The experimental results show that the proposed method can be used to calculate clinical gait parameters by estimating foot trajectory.

The proposed gait analysis method comprises two IMUs with a triaxial accelerometer, triaxial gyroscope, and tablet computer. This method can be applied in a variety of locations outside of the gait laboratory and is less expensive than conventional gait analysis methods such as motion capture systems. The clinical advantage is that the patient burden is low because of the light weight (about 24 g) and easy attachment of the IMUs. We therefore anticipate that the proposed method would be suitable for clinical gait analysis. As for the location of the IMUs, the *R* value between displacement in the direction of forward movement as measured with the marker attached on the IMU and as measured with the marker attached on the heel (0.994) indicates that the location of the IMUs is valid at least for estimating the stride length. The *R* value between displacement in the direction of forward movement as estimated by the proposed method and as measured with the marker of the motion capture system attached on the IMU indicates that displacement in the direction of forward movement estimated by the proposed method explained 96% of the variation in displacement in the direction of forward movement as measured with the motion capture system.

The mean error of stride length estimated with the proposed method was 0.054 ± 0.031 m (Table 1). This result suggests that the proposed method can estimate clinical gait parameters such as stride length. A previous method in which the location of a IMU is on the dorsum of a foot found that the accuracy mean accuracy ± precision was 0.015 ± 0.068 m (Mariani et al., 2010). The IMU location on the shank may cause bias of this order of accuracy. We expected that further development of the method will overcome this limitation of performance. Several studies (Stolze et al., 2001; Curtze et al., 2015) have found that stride length is shorter in patients with PD than it is in healthy controls as observed in the example of the application of the proposed method (Table 2). For example, Morris et al. (2005) reported that stride length in PD patients in the off state was 0.96 ± 0.19 m, which was shorter than the stride length of 1.46 ± 0.08 m as measured in healthy age-matched controls. The *R* value between displacement in the direction of forward movement estimated by the proposed method and measured with the marker of the motion capture system attached on the heel indicates that stride length estimated by the proposed method explained 96% of the variation measured with the motion capture system. This result suggests that IMUs are potentially useful in clinical gait analysis. We expect that further development of the proposed method to evaluate the gait in people with PD.

We expect that further development of this method or other methods will enable us to evaluate quantitatively the effects of drugs and interventions such as rehabilitation in patients with gait disorders. In the future, we plan to assess patients with gait abnormalities, such as those caused by PD. We will validate the proposed method to determine whether it can identify abnormal gait patterns, including shuffle, short-steppage, and hemiplegic gaits. The sampling frequency that we used in the present study was 100 Hz. We plan to investigate the effect of the sampling frequency on the estimation of the gait parameter in the next study.

## Conclusion

Our results suggest that the proposed method is suitable for gait analysis whereas there is a room for improvement of its accuracy. Unlike methods that use motion capture systems, this method can be used in a variety of locations, such as in the corridor of a medical center. Further development of our proposed method is expected to enable clinicians to share objective information about gait features with health-care providers and patients.

## Data Availability Statement

The datasets for this article are not made publicly available because there is an agreement for data exchange between Tokyo Institute of Technology and Kanto Central Hospital.

## Ethics Statement

The Ethics Committee of Tokyo Institute of Technology approved the protocols for this study, and all participants provided written informed consent.

## Author Contributions

KH, YO, YH, YMa, HO, SO, and YMi designed the research. YO, KH, YH, HS, and AI performed the experiments. YMa, YO, HO, KH, and YMi analyzed the data. HO, YO, and YMi wrote the paper.

### Conflict of Interest

The authors declare that the research was conducted in the absence of any commercial or financial relationships that could be construed as a potential conflict of interest.
